# Lateral Condyle Fracture of the Humerus in the Pediatric Age Group: A 10-Year Tertiary Center Experience

**DOI:** 10.7759/cureus.78180

**Published:** 2025-01-29

**Authors:** Abdulmalek I Alnujidi, Abdulrahman H Almalki, Omar Almutair, Shahid A Abak, Nawaf N Alamri

**Affiliations:** 1 Orthopedic Surgery, Prince Sultan Military Medical City, Riyadh, SAU; 2 Orthopedics, King Abdulaziz Medical City, Riyadh, SAU; 3 Reconstructive Orthopedics, King Fahad Medical City, Riyadh, SAU; 4 Pediatric Orthopedics, King Abdullah Specialist Children Hospital, Riyadh, SAU

**Keywords:** avascular necrosis, cubitus valgus deformity, cubitus varus, delayed union, elbow fractures, lateral condyle, lateral condyle overgrowth

## Abstract

Objective

Lateral humeral condyle fracture is a relatively common elbow injury among children, which often has a subtle presentation and a high risk of developing complications. This study aimed to discuss the epidemiology of such injury among children presenting to a tertiary hospital emergency room in Riyadh, Saudi Arabia. Other objectives included analyzing possible predictors and risk factors for developing complications in such patients, which will contribute to implementing preventive measures.

Methodology

This was a single-center, retrospective study conducted on data collected from patients presenting to the pediatric emergency department of a tertiary hospital in Riyadh, Saudi Arabia, from January 1, 2014, to July 2024. Patients under 18 years of age, with a minimum of six months of follow-up and complete data in our medical records, were included. A total of 77 patients were identified by convenience sampling; seven were excluded due to incomplete data or a follow-up period of less than six months. Where appropriate, statistical significance between variables was assessed using Fisher’s exact test and Pearson’s chi-square test. A *P*-value cutoff of 0.05 at a 95% confidence interval was used to determine statistical significance.

Results

This study reviewed 70 cases of lateral condyle fractures of the humerus. The mean age of the patients was 4.34 years, with 42 (60%) being under five years. The majority of patients (36, 51.4%) were male, and 42 (60%) sustained their injuries at home. Most patients (51, 72.78%) presented with types 2 and 3 displacements. Initial management for the majority of patients (52, 74.3%) was surgical. Of the entire sample, 40 (57%) patients had complications, with lateral condyle overgrowth being the most common, followed by cubitus varus and elbow stiffness. Females were found to present with significantly less displacement (*P* = 0.037). The degree of displacement was found to be a strong predictor of developing fracture nonunion (*P *= 0.0392) and lateral condyle overgrowth (*P* = 0.02).

Conclusions

Our study demonstrates that lateral condyle fractures are associated with a high rate of complications and that surgical intervention is often required for such injuries. A high index of suspicion and physician vigilance is essential, as this injury can be easily missed if it is not displaced. Prompt identification and management are crucial to reduce the risk of subsequent complications.

## Introduction

Fractures of the lateral condyle of the humerus are one of the most common elbow injuries among children, representing around 10%-20% of all pediatric elbow fractures [1]. There are two hypothesized mechanisms for the occurrence of this injury: either direct force from the radial head on the condyle after a fall on an outstretched hand, or avulsion forces from the common extensor origin [2]. Lateral condyle fractures can be subtle and difficult to visualize on plain radiographs, which can be attributed to the cartilaginous nature of the capitellum in young children. This makes it challenging to diagnose and assess displacement upon initial presentation to the emergency room (ER), making subsequent follow-up radiographs essential to rule out fractures [3].

Patients with lateral condyle fractures commonly present with lateral elbow pain and a limited range of motion at the elbow. Obvious deformity and swelling are not usually seen among patients presenting with this condition, so a high index of suspicion is necessary to perform elbow radiographs for these patients [4]. Several classification systems have been developed to guide treatment. Milch classified lateral condyle fractures into two types: (1) Type 1 fracture, where the fracture line passes just lateral to the trochlear groove. These fractures are believed to leave the elbow stable, and (2) Type 2 fractures, which extend into the trochlear groove or just medial to it. These fractures are associated with a high rate of elbow instability [5,6]. A more recent classification system was introduced by Weiss et al., where fractures were grouped based on their displacement and followed for short-term and long-term complications. Type 1 fractures indicated less than 2 mm displacement, Type 3 fractures indicated over 4 mm displacement, and Type 2 fractures were in between Types 1 and 3. This classification system had the advantage of being predictive of future complications, making it more clinically useful [7]. In minimally displaced fractures (displacement <2 mm), conservative management with long-arm casting is recommended. When casting, the forearm should be kept in supination and the wrist extended to reduce the muscle pull of the supinator-extensor muscle complex attached to the lateral condyle, thereby reducing subsequent displacement [8].

Open fractures and non-reducible fractures are absolute indications for surgical management [9]. Another indication for surgical management is moderate to severe displacement, which is defined as displacement greater than 2 mm [10]. There are multiple complications associated with lateral condyle fractures, such as delayed union, malunion, pseudoarthrosis, cubitus valgus deformity, tardy ulnar nerve palsy, radial elbow instability, fishtail deformity, and cosmetic impairment [11]. Another complication is the development of an abnormal carrying angle caused by overgrowth of the lateral condyle. This abnormality is considered the most common growth disturbance following a lateral condyle fracture [12].

## Materials and methods

Study design and setting

This was a quantitative, cross-sectional, single-center retrospective study carried out on the data collected from patients presenting to the pediatric emergency department at a tertiary hospital in Riyadh, Saudi Arabia, from January 2014 to July 2024.

Inclusion and exclusion criteria

Patients aged 18 years or younger, presenting with an isolated or combined lateral condyle fracture, with complete data in the system and a minimum follow-up of six months, were included in the study. A total of 77 patients were originally identified; 70 were included and seven were excluded: three sustained their injuries recently and did not meet the follow-up criteria of six months, two had missing details in their medical records, and two were actually cases of supracondylar humeral fractures that were wrongly labeled as lateral condyle fractures.

Data collection

The data collected were divided into two parts. Part 1 included demographic data, such as age, sex, and the setting where the injury occurred (Table 1). Part 2 included the following details: (1) mode of injury (fall on outstretched hand (FOOSH) vs. direct blow to the elbow); (2) type of fracture (open vs. closed); (3) degree of displacement (Type 1, <2 mm; Type 2, between 2 and 4 mm; Type 3, >4 mm); (4) concurrent musculoskeletal injuries; (5) whether the fracture was missed at the initial presentation; (6) distal neurovascular integrity, including any vascular injuries or nerve palsy; (7) initial management (long arm cast immobilization vs. surgical intervention); (8) whether surgical intervention was ultimately necessary (this question applies only to patients who were treated conservatively); and (9) occurrence of complications such as malunion, nonunion, growth disturbances, and nerve palsy up to six months after injury.

**Table 1:** Patient demographic characteristics (n = 70). SD, standard deviation

Study variables	*n* (%)
Age in years (mean ± SD)	4.34 ± 2.32
<5	42 (60.0%)
≥5	28 (40.0%)
Gender	
Male	36 (51.4%)
Female	34 (48.6%)
Place of injury	
Home	42 (60.0%)
Recreational	22 (31.4%)
School	06 (08.6%)

Sampling technique and statistical analysis

Due to the uncommon incidence of the injury, the sampling technique was convenience sampling to obtain as many subjects as possible, thus yielding more generalizable results. Frequency and proportion were used to summarize all categorical variables in this project, while mean, median, and standard deviation (SD; min-max) were used to present all continuous variables. Statistical significance between variables was measured by using Fischer’s exact test and Pearson’s chi-square test, where appropriate. A *P*-value cutoff point of 0.05 at a 95% confidence interval (CI) was used to determine statistical significance. The development of complications was the main outcome variable tested; each complication was then tested separately. All data analyses were performed using SPSS, version 26 (IBM Corp., Armonk, NY).

Ethical considerations

Institutional Review Board approval from King Abdulaziz City for Science and Technology (H-01-R-012) was obtained before data collection. Datasheets were coded to ensure patient anonymity. Data were entered into a secure PC, and only investigators had access to data during the period of the study.

## Results

**Figure 1:**
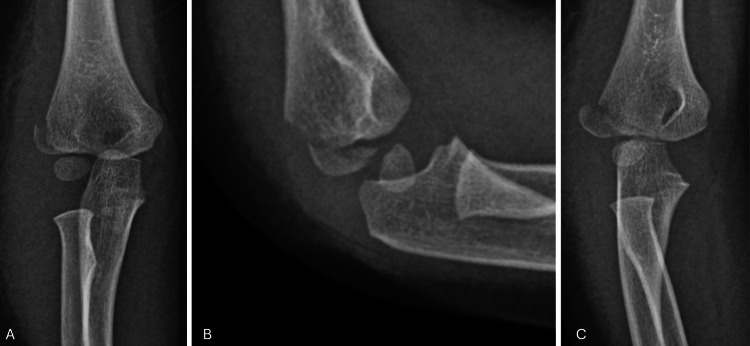
(A) Anteroposterior (AP), (B) lateral, and (C) oblique views of right elbow radiographs. AP = anteroposterior.

This study involved 70 cases of lateral condyle fractures of the humerus. Figure 1 shows the presenting radiographs of one of the subjects. As demonstrated in Table 1, the mean age was 4.34 (SD 2.32) years, with 42 patients (60%) younger than five years. More than half (36, 51.4%) were boys, and 42 (60%) sustained their injuries at home.

**Figure 2:**
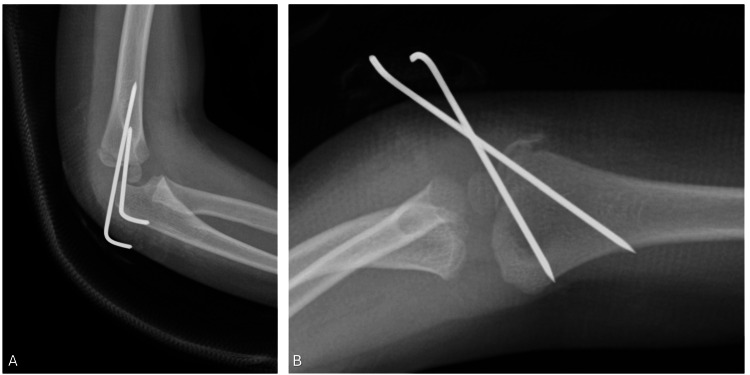
(A) Anteroposterior (AP) and (B) lateral views of the right elbow. Images were taken immediately after open reduction and Kirschner wire, or K-wire, fixation.

As shown in Table 2, the most common mode of injury was FOOSH (56, 80%). The majority of the patients (68, 97.1%) presented with closed fractures. Nearly half of the sample (32, 45.7%) had Type 3 (>4 mm displacement). Most of the patients (61, 87.1%) had no concurrent injuries in the elbow; supracondylar fractures were most common (4, 5.7%). We also noted that eight (11.4%) of the fractures were missed at the initial presentation. All patients had intact distal neurovascular functions. Initial management was mostly surgical in 52 (74.3%) patients; 32 patients (61.5%) had closed procedures in the form of closed reduction and percutaneous pinning, while closed reduction was not possible in the remaining 20 (38.5%) patients. Open reduction and pinning were performed, as shown in Figure 2. Conservative treatment was opted in for 18 patients (25.7%) - none had fracture displacement on follow-up and none needed surgical intervention.

**Table 2:** Injury characteristics, type of management, and complications. ^*^Some patients had more the one complication simultaneously. FOOSH, fall on outstretched hand

Variables	n (%)
Mode of injury	
FOOSH	56 (80.0%)
Direct blow to the elbow	14 (20.0%)
Type of injury	
Open	02 (02.9%)
Closed	68 (97.1%)
Fracture displacement	
Non-displaced or displaced less than 2 mm (Weiss et al. Type 1)	19 (27.1%)
Minimally displaced (2-4 mm) (Weiss et al. Type 2)	19 (27.1%)
Displaced more than 4 mm (Weiss et al. Type 3)	32 (45.7%)
Concurrent injuries in the elbow	
None	61 (87.1%)
Supracondylar fracture	04 (05.7%)
Distal radius fracture	02 (02.9%)
Olecranon fracture	03 (04.3%)
Whether fracture was missed at the initial presentation	
Yes	08 (11.4%)
No	62 (88.6%)
Intact distal neurovascular status	
Yes	70 (100%)
No	0
Initial management	
Conservative	18 (25.7%)
Surgical	52 (74.3%)
Whether the fracture fragment had displacement on follow-up if initial management was conservative (n = 18)	
Yes	0
No	18 (100%)
Whether surgical intervention was needed ultimately (n = 18)	
Yes	0
No	18 (100%)
Whether the surgical intervention was open or closed (n = 52)	
Open	20 (38.5%)
Closed	32 (61.5%)
Complications^*^	
None	30 (42.85%)
Varus deformity	12 (17.14%)
Lateral condyle overgrowth	15 (21.4%)
Surgical site infection	2 (2.85%)
Elbow stiffness	10 (14.3%)
Nonunion/delayed union	4 (5.71%)
Valgus deformity	2 (2.85%)

**Figure 3:**
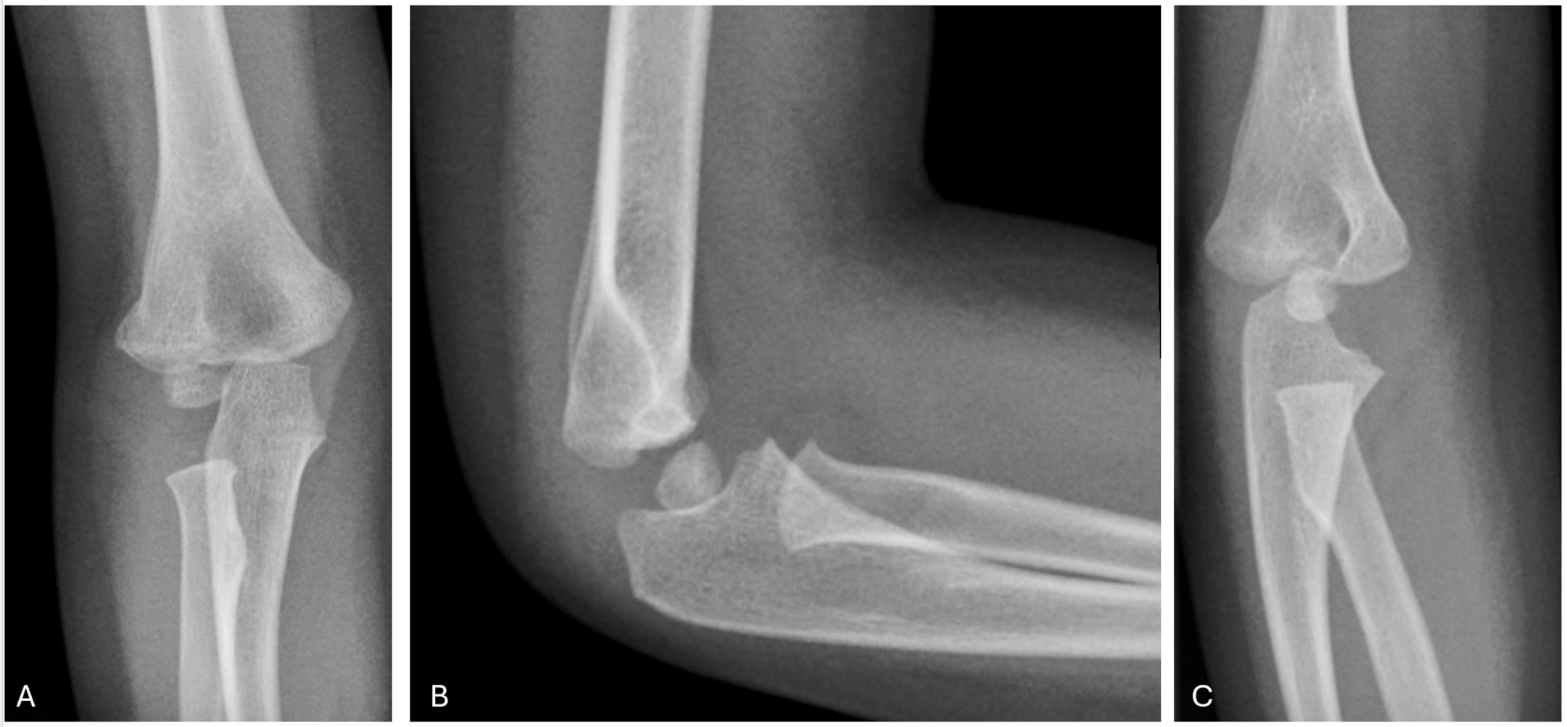
Images taken three months postoperative show (A) anteroposterior (AP), (B) lateral, and (C) oblique views, with complete fracture healing. K-wires were removed in the clinic six weeks postoperative.

Of the entire sample, 30 (43%) achieved complete fracture union with no complications (Figure 3). However, 40 (57%) had complications. Lateral condyle overgrowth (15, 21.4%) was the most common complication, followed by cubitus varus (12, 17.4%) and elbow stiffness (10, 14.3%). The least reported complications were surgical site infections (SSIs) and cubitus valgus, with two cases each (2.85%).

When comparing gender to patient demographic characteristics, injury, management, and complications, as shown in Table 3, it was observed that girls (*P* = 0.037) were less likely to present with a higher degree of fracture displacement than boys. No significant differences were observed between boys and girls in relation to age, place of injury, mode of injury, type of injury, concurrent injuries in the elbow, fracture missed in the initial presentation, initial management, and complications (all *P* > 0.05).

**Table 3:** Gender differences in relation to fracture displacement. ^§^*P*-value calculated using Fischer’s exact test. ^*^Significant at *P* < 0.05, at 95% CI. CI, confidence interval

Factor	Boys, *n* (%) (*n* = 36)	Girls, *n* (%) (*n* = 34)	*P*-value^§^	Fisher-Freeman-Halton exact test
Fracture displacement				
Non-displaced or displaced (<2 mm)	06 (16.7%)	13 (38.2%)	0.037^*^	6.702
Minimally displaced (2-4 mm)	14 (38.9%)	05 (14.7%)		
Displaced (>4 mm)	16 (44.4%)	16 (47.1%)		

As shown in Table 4, a higher degree of displacement was associated with a higher rate of delayed union or nonunion of the fracture (*P* = 0.039), as well as a higher rate of lateral condyle overgrowth (*P* = 0.02).

**Table 4:** Rate of nonunion and lateral condyle overgrowth in relation to fracture displacement. ^§^*P*-value calculated using Pearson’s chi-square test. ^*^Significant at *P* < 0.05, at 95% CI. CI, confidence interval

Complication	Non-displaced or minimally displaced	Displaced more than 4 mm	*P*-value^§^	Pearson chi-square
None (*n* = 30)	20 (66.6%)	10 (33.3%)	N/A	N/A
Nonunion (*n* = 4)	0 (0.0%)	4 (100%)	0.0392*	5.038
Lateral condyle overgrowth (*n* = 15)	4 (26.7%)	11 (73.3%)	0.02*	5.87

## Discussion

The primary objective of this study was to discuss the epidemiology of lateral condyle fractures, including injury patterns, management options, and complications in patients aged 0-18 years. Secondary objectives included analyzing possible predictors and risk factors of complications in patients presenting with such injuries. Lateral condyle fracture is not an uncommon injury, with associated serious complications such as nonunion, malunion, avascular necrosis of the lateral condyle, and neurovascular injury [3]. A higher rate of injury was found among boys, which has also been noted in the literature [13]. The mean age of injury was approximately 4.32 years, which is similar to the age of five years reported in another study [14]. No differences in prevalence were noted among both genders, which is consistent with that in the literature [1]. Supracondylar fractures were the most reported associated injury, attributable to the fact that both fractures can happen as a result of FOOSH. Olecranon fractures and distal radial fractures were the second and third most presenting associated injuries. Associated injury to the ipsilateral upper limb was found to be associated with poorer outcomes, as reported by Sharma et al. [15].

Although we noted a higher percentage of complications among patients with concomitant elbow injuries, no statistically significant results were reported. Non-displaced fractures (<2 mm displacement) are often managed conservatively with cast immobilization, yielding excellent results, as reported by Foster et al. [16] and Mintzer et al. [17]. In a recent systemic review, Knapik et al. analyzed six studies, following non-displaced or minimally displaced lateral condyle fractures that were treated conservatively [18]. They reported a rate of interval displacement on follow-up radiographs as high as 14.9% among 355 patients, with an average displacement ranging from 1.3 to 2.5 mm, which eventually necessitated surgical intervention. Secondary displacement was found to be likely to occur within the first week, or as early as the first three days post-splinting, as reported by Finnbogason et al. [19], which is relatively similar to findings reported by Pirker et al. [20]. These findings emphasize the need for close follow-up and repeat radiographs within the first week of injury as a standard of care [3].

Among our sample, 18 patients (25.7%) were treated with long-arm cast immobilization, of which 15 had non-displaced fractures. Three patients in the minimally displaced group were also treated with cast immobilization. No secondary displacement was noted among both groups. Of our patients, eight (11.4%) were missed on presentation to the ER and were identified by either visiting the ER again for persistent pain or by clinic referrals. This is a higher rate than that of 5.2% reported by James et al. [3]. Non-displaced or minimally displaced fractures are sometimes difficult to detect by the untrained eye, so many ER physicians would consider AP and lateral views to suffice for diagnosis because the general rule is to request at least two orthogonal views to diagnose a fracture. However, this is not the case in elbow trauma where oblique views are mandatory [21]. Providing further training to ER residents and fellows as well as having a high index of suspicion to obtain orthopedics’ or radiologists’ input before patients’ discharge will help reduce the rate of missed injuries. This is of paramount importance because delayed diagnosis will lead to further fracture displacement, which leads to serious complications such as nonunion [22].

Of the 52 patients who underwent surgical intervention, only two experienced SSIs. Both patients presented with open fractures, which is a well-known risk factor for SSI. Lateral condyle fractures carry a high risk of complications. In our sample, 39 (55.7%) had experienced one or more complications. In our study, lateral condyle overgrowth was the most reported complication. This is similar to what is reported in the literature, reaching as high as 73%, varying from mild to moderate and severe [23]. Initial displacement and open surgical intervention are both considered well-known risk factors for the development of such a complication [24]. However, Meng et al. published a meta-analysis comparing both closed reduction percutaneous pinning and open reduction and found no significant relationship between both approaches about the risk of developing lateral condyle overgrowth [25]. Nonunion, defined as no signs of healing on radiographs at eight weeks post-injury, is a classic complication of lateral condyle fractures. Four patients (5.71%) experienced delayed union or nonunion. Few authors examined the rate of nonunion in such fractures, ranging from as little as 1.4% to 5%, to as high as 16%. The presence of a Type 3 fracture was found to be a statistically significant risk factor for the development of delayed union or nonunion, which is in agreement with the current literature (Table 4) [15,26,27]. The remaining complications were more common among groups with a higher degree of fracture displacement. However, none was statistically significant.

Lateral condyle fracture management aims to anatomically restore articular surface congruency. There is a consensus that fracture displacement of more than 2 mm, as seen in Figure 1, and articular step-off and incongruency are absolute surgical indications for intervention [28]. Surgical options include closed reduction and percutaneous pinning, which usually suffice in Weiss Type 2 and in cases where the articular hinge is intact [7]. In a closed setting, an elbow arthrogram can be used to assess reduction and confirm anatomical articular reduction. Other options include open reduction and internal fixation (Figure 2), which are reserved for Weiss Type 3 fractures and irreducible fractures by closed means [28]. Methods of fixation include K-wires and cannulated screw fixation. K-wires have shown excellent results and a complete union of 105 patients in a study published by Leonidou et al. [29]. No significant differences were reported in the development of subsequent complications when comparing both methods [30].

More generalizable results could have been achieved with the probability sampling technique. However, due to the limited number of patients presenting with such an injury, we opted for sampling by convenience. A bigger sample size could have been achieved by making this a multicenter study. Due to the design of the study, it was subjected to biases typical of retrospective studies. To our knowledge, no local studies were published to examine the epidemiology of lateral condyle fractures in Saudi Arabia, imposing another limitation on the study.

## Conclusions

Lateral condyle fractures are relatively common elbow injuries that require careful clinical assessment for timely diagnosis. Repeat radiographs within three to five days are recommended to identify any missed injuries. In our study, most cases were managed according to the Weiss et al. classification, which has proven effective in predicting subsequent complications.

Although rare, complications such as nonunion and malunion can occur, particularly in fractures with significant fragment displacement. These complications are associated with a higher risk of long-term functional impairment. Effective management and early intervention are crucial in preventing these outcomes and essential to minimize both patient morbidity and healthcare costs.
